# Near-infrared guided robotic partial cystectomy for urachal adenocarcinoma using the da Vinci Xi Firefly platform

**DOI:** 10.3389/fsurg.2026.1916543

**Published:** 2026-08-27

**Authors:** L. Isherwood, M. Feyissa, M. Muheilan, K. Thandapani, N. Vasdev

**Affiliations:** Urology Department, East and North Hertfordshire Teaching NHS Trust, Stevenage, United Kingdom

**Keywords:** bladder cancer, firefly, near-infrared, partial cystectomy, robotic partial cystectomy, urachal adenocarcinoma

## Abstract

Partial cystectomy is considered an optimal bladder-preserving surgical treatment for patients with adenocarcinoma confined to the urachus. This case report describes the use of intraoperative near-infrared fluorescence imaging to fully resect a urachal adenocarcinoma in a partial cystectomy and ensure clear oncological margins while preserving bladder function. The patient described was a male in his 50s who presented to the emergency department with visible haematuria. Both CT and MRI imaging, combined with transurethral resection of the lesion, confirmed a urachal adenocarcinoma. The patient was scheduled for a near-infrared-guided robotic partial cystectomy and pelvic lymph node dissection using Firefly imaging and intraoperative transurethral resection of the bladder tumour. During the procedure, Firefly imaging on the da Vinci Xi robot, combined with cystoscopic transillumination of the tumour, enhanced the surgeon's ability to confidently resect the tumour while sparing the adjacent healthy bladder tissue. The patient recovered well postoperatively and was discharged from hospital after 48 h. A CT cystogram after 4 weeks confirmed that the bladder defect was closed and that the patient was able to void urine without a catheter. Histological analysis confirmed that the tumour had been fully resected and that all pelvic lymph nodes were clear of metastatic disease; however, muscle invasion was present (pT2 disease). This case demonstrates the feasibility of performing a near-infrared-guided robotic partial cystectomy for urachal adenocarcinoma, offering surgeons the ability to confidently resect urachal tumours in a partial cystectomy by providing clear oncological margins and preserving bladder function.

## Background

Adenocarcinoma of the bladder is a very rare subtype of bladder cancer that constitutes 0.5%–2% of total bladder cancer diagnoses (1). Radical cystectomy offers a definitive cure in these cases; however, advancements in urological surgery have led to surgeons being able to offer various types of bladder-preserving procedures for the management of bladder cancer. For a select subgroup of patients with isolated urachal adenocarcinoma, partial cystectomy is a procedure with potentially curative outcomes (2). A partial cystectomy enables surgeons to remove a tumour while sparing the bladder, which offers the patient optimal outcomes and a better quality of life compared to a radical cystectomy (2). The challenges of performing partial cystectomies in these cases include preserving bladder capacity when large resections are required and identifying tumour margins, which can lead to positive margins and disease recurrence. Near-infrared imaging is a surgical technique that has been adopted for other urological surgeries to intraoperatively identify tumour margins and can be used as an adjunct in partial cystectomies to aid identification of the edge of a bladder tumour during dissection (3). Near-infrared light has a wavelength of 700–900 nm and is therefore not visible to the human eye; however, the Firefly setting on the da Vinci Xi platform (Intuitive Surgical, CA, USA) can be used to penetrate tissues deeper than visible light can, allowing deep structures to be visualised with a green colour (4, 5). This surgical technique is not extensively documented in the surgical literature, and therefore, this case report serves as a review of this emerging and novel technique for performing partial cystectomies using near-infrared guidance.

## Case presentation

**Figure 1:**
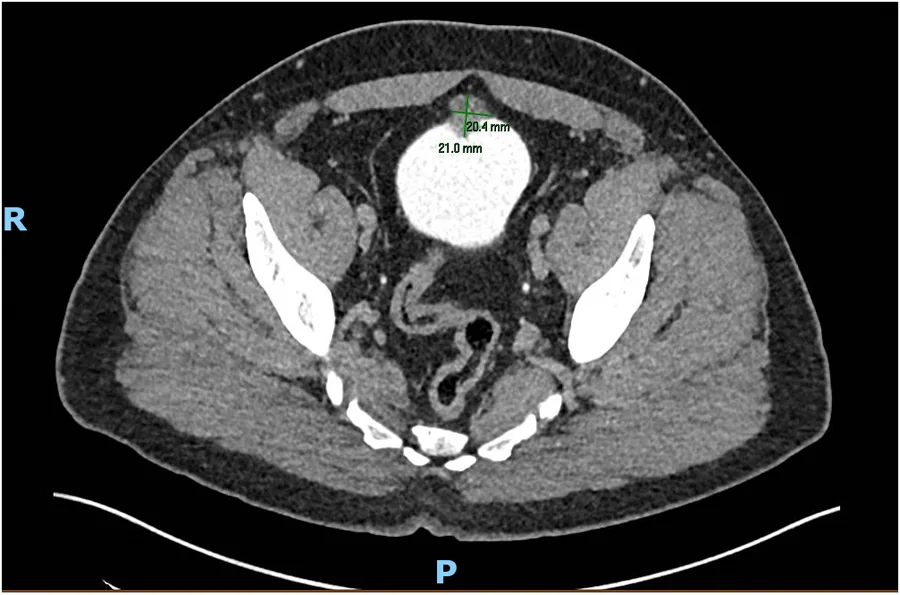
A CT urogram shows a polypoid lesion at the bladder dome (transverse plane).

A male patient in his 50s presented to the emergency department with an episode of visible haematuria with clots (Table 1). He was subsequently followed up on the urological cancer pathway in an outpatient clinic for his haematuria, where he underwent a flexible cystoscopy, which showed a 3-cm polypoid lesion at the dome of the bladder (Figure 1). A CT urogram similarly confirmed a polypoid growth originating from the anterior and superior walls of the bladder. Differential diagnoses at this stage included urothelial carcinoma with glandular differentiation, primary bladder adenocarcinoma, and secondary adenocarcinoma, e.g., colorectal in origin.

**Table 1:** Clinicopathological and prognostic information.

Clinicopathological/prognostic information	Details
Presenting symptoms	Visible haematuria
Imaging	CT urogram: polypoid growth arising from the anterior and superior wall of the bladder
	Bladder MRI: Urachal cystic solid mass
Cystoscopy	3-cm polypoid lesion at the dome of the bladder
Surgery	TURBT: Features of adenocarcinoma. Differential diagnoses include urachal tumour, primary bladder adenocarcinoma, primary colorectal carcinoma, and metastasis
	Near-infrared-guided robotic partial cystectomy and pelvic lymph node dissection using Firefly imaging and intraoperative transurethral resection of the bladder tumour
Histopathology and immunohistochemistry	20-mm primary muscle-invasive (pT2) bladder adenocarcinoma with moderately differentiated features
	Diffuse staining with CEA and CDX2 markers.
	Scattered staining with CK20 marker
	Diffuse p53 staining suggesting an aggressive phenotype
Stage	Primary muscle-invasive (pT2) bladder adenocarcinoma
	Sheldon stage IIIA disease
Lymph nodes	0/6 positive lymph nodes
Postoperative course	Discharged 2 days postoperatively
	Catheter removed 4 weeks postoperatively, with CT cystogram confirming no leak and the patient continent
	Long-term management for stage IIIA disease yet to be confirmed

TURBT, transurethral resection of bladder tumour.

Subsequent transurethral resection of the bladder tumour was performed alongside a contrast MRI scan of the bladder, which was discussed at a multi-disciplinary meeting at the lead urological cancer centre in the region to confirm the diagnosis of a urachal cystic solid mass with primary adenocarcinoma on histology. A decision was made to proceed with a near-infrared-guided robotic partial cystectomy and pelvic lymph node dissection using Firefly imaging and intraoperative transurethral resection of the bladder tumour. The patient was fit and well, with an American Society of Anesthesiologists (ASA) level of II and had no other past medical history other than bladder adenocarcinoma. He was an ex-smoker with a six pack-year history, drank 15 units of alcohol per week, and had no past medical history or family history of bladder or renal cancer.

## Treatment

### Surgical procedure

The patient underwent a robotic partial cystectomy and pelvic lymph node dissection using near-infrared fluorescence guidance 8 weeks after their initial attendance at the urology clinic. The procedure was performed with a pneumoperitoneum of 8 mmHg, as this lower pressure has been demonstrated to reduce intraoperative and postoperative complications in patients and subsequently improve clinical outcomes (6).

**Figure 2:**
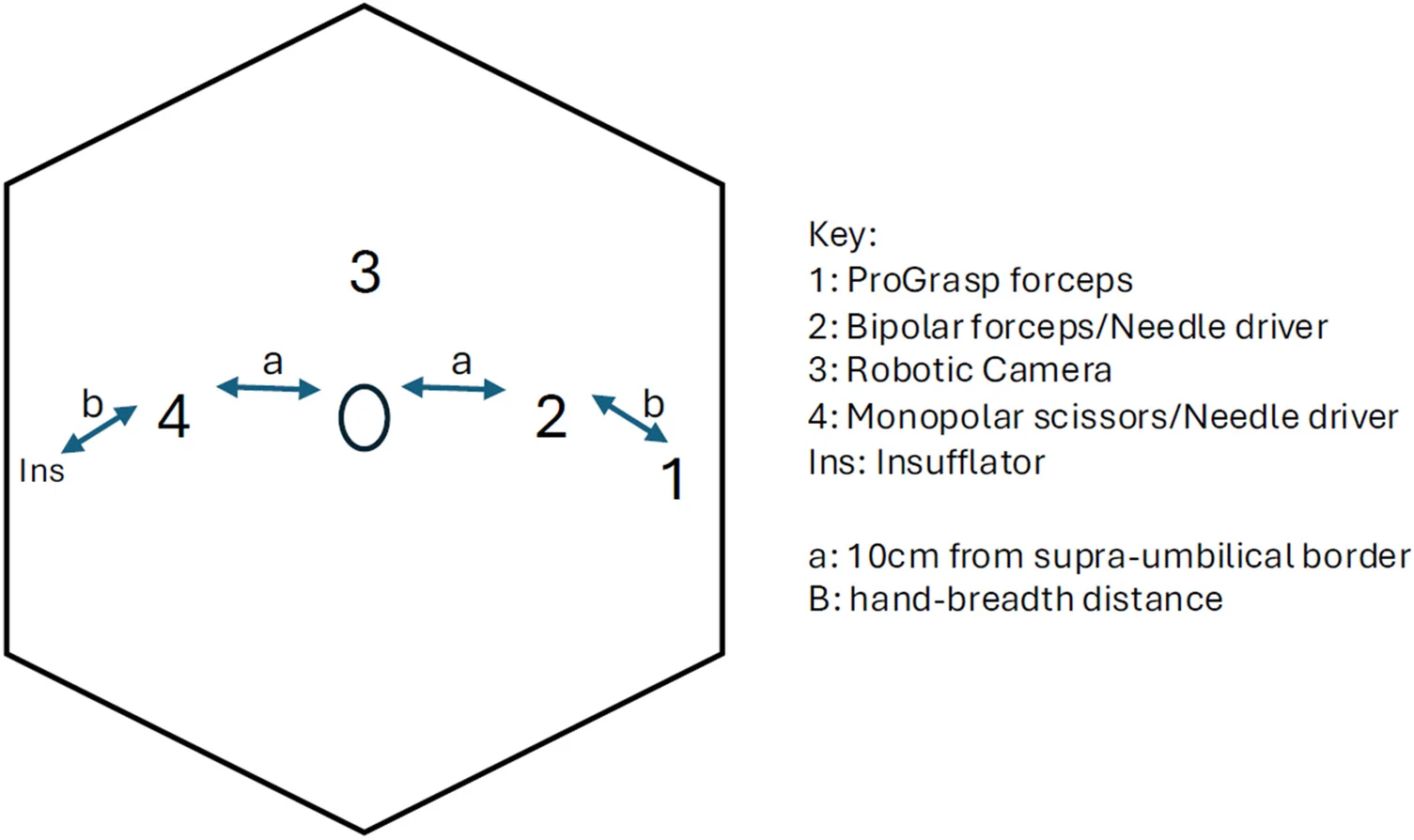
A schematic diagram of the port placement for the partial cystectomy.

At induction, the patient was administered IV gentamicin 120 mg, co-amoxiclav 1.2 g, and metronidazole 500 mg, and was placed in the lithotomy position. Rigid cystoscopy was performed to identify the 3 cm tumour at the bladder dome, and the outline of the lesion was marked using a Collin's knife via a resectoscope, leaving a 1 cm margin. Once the tumour outline was demarcated, a 22 French three-way catheter was inserted, and saline irrigation was commenced. The patient was subsequently placed in the steep Trendelenburg position to mobilise the small bowel out of the operative field. The initial supraumbilical robotic port was inserted using the Hasson technique and a pneumoperitoneum of 8 mmHg was achieved. Three 8-mm robotic ports, one 12-mm SurgiQuest AirSeal® (CT, USA) insufflator port, and one 5-mm assistant port were placed *in situ,* and the da Vinci Xi robot was docked (Figure 2). The valveless design of the AirSeal insufflator has been shown to have superior smoke evacuation and pneumoperitoneum stability compared to conventional valved insufflators, and provides improved surgical visualisation and reduces intraoperative bleeding (6). Initially, the urachus was divided, and the bladder was lowered using lateral mobilisation by releasing the endopelvic fascia. This process allowed for easier manipulation of the bladder during dissection of the bladder tumour. The extensive adhesions that were present between the bladder and the sigmoid colon were released using an athermal technique.

**Figure 3:**
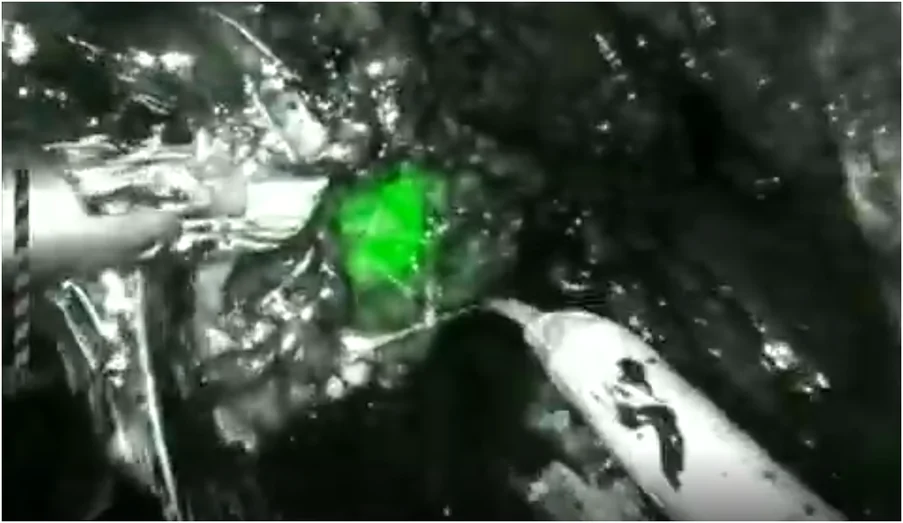
Near-infrared Firefly imaging shows the tumour margin, transilluminated with a flexible cystoscope.

**Figure 4:**
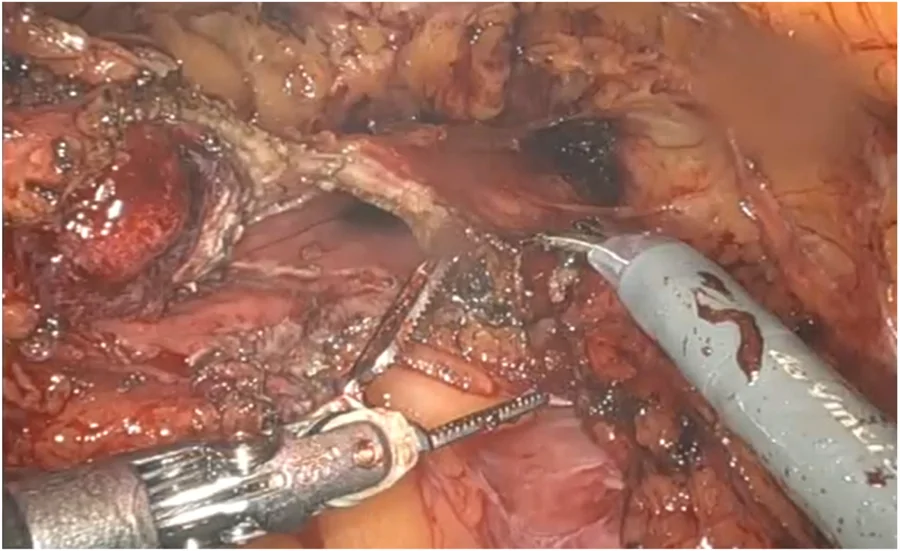
Dissection around the tumour margins identified by near-infrared imaging.

The three-way catheter was subsequently removed, and a flexible cystoscope was introduced into the bladder to intravesically visualise the tumour and the pre-resected tumour margins. While the flexible cystoscope was used to transilluminate the bladder tumour margins, the Firefly mode on the da Vinci Xi robot, using near-infrared waveforms, allowed light penetration into deeper tissues, thus allowing the surgeon to accurately and safely dissect around the margin of the bladder tumour. Instead of estimating the location of the bladder tumour during partial cystectomy, this technique allowed the surgeon to directly visualise the tumour, meaning tumour margins were not compromised, and ensured excess bladder tissue was not resected (Figures 3, 4). Therefore, postoperative bladder capacity was not compromised. The bladder tumour at the dome was meticulously dissected from adjacent healthy bladder tissue and the urachus. Extravesical fat was also removed and placed inside an Endo Catch™ bag (Medtronic, MN, USA). No intraoperative frozen section histology was used. The bladder defect from this partial cystectomy was closed in three layers using 3-0 V-Loc™ (Medtronic, MN, USA) sutures. A three-way catheter was reinserted, and a leak test of the bladder using 120 mL of saline was performed to ensure the bladder defect was adequately closed.

Additionally, a level 2 pelvic lymph node dissection was performed, which involved clearance of the internal and external iliac nodes. The surgeon was meticulous in identifying and preserving the obturator nerves bilaterally. Removal of the pelvic lymph nodes offered the optimal oncological resection for this patient.

At the end of the procedure, a 20 French Robinson drain was left inside the pelvis via the left robotic port. This drain is beneficial in the postoperative period as it can aid in the early detection of postoperative complications such as bleeding or a urine leak. Upon removal of the 12-mm AirSeal port, the peritoneal defect was closed robotically using 2-0 Vicryl. The robot was undocked from the remaining ports, and the specimen was extracted via the defect left of the midline supraumbilical port. Midline wound closure was done with 1 loop of PDS (polydioxanone, Ethicon, NJ, USA) and reinforced with 2-0 Ethibond (Ethicon, NJ, USA). All the ports had the subcutaneous layer closed with 2-0 Vicryl, and the skin edges were opposed with staples.

Total blood loss was 20 mL. The total operating time was 170 min, with a console time of 100 min.

Postoperative instructions included keeping the three-way catheter *in situ* on free drainage with slow saline irrigation for 24 h to reduce postoperative haematuria and prevent clot retention. After 48 h, the three-way catheter was switched to a two-way Foley catheter, which remained in place for 4 weeks until a CT cystogram confirmed no urinary extravasation secondary to a defect in the closure of the bladder wall. The patient was administered 28 days of enoxaparin 40 mg SC OD on day 2 post-operation, 14 days of treatment-dose oral co-amoxiclav, 28 days of prophylactic-dose nitrofurantoin, 14 days of anti-embolic stockings, and 14 days of lactulose.

## Outcome and follow-up

The patient had an optimal recovery and did not require any higher-level care on intensive care unit or high dependency unit. The patient's post-op bloods were all within normal range other than a mildly elevated white cell count (13.2 × 10^9^/L) and C-reactive protein level (73.7 mg/L), which are to be expected following surgery. The patient's creatinine level remained the same as his pre-op level (90 µmol/L), and his haemoglobin dropped by 26 g/L from 164 to 138 g/L.

The postoperative histological analysis confirmed a 20-mm primary muscle-invasive (pT2) bladder adenocarcinoma with moderately differentiated features, characterising it as Sheldon stage IIIA disease. On immunohistochemistry, there was staining with carcinoembryonic antigen, caudal type homeobox 2, and cytokeratin 20 markers and p53 staining, which indicated an aggressive disease phenotype. The resection margins were clear by 3 mm, and no tumour deposits were seen in the soft tissues covering the urachus or bladder. No lymphovascular invasion was present, and none of the six pelvic lymph nodes dissected showed any evidence of tumour metastasis. No long-term follow-up data on the patient's outcomes have been collected, and the postoperative adjuvant management plan has yet to be decided.

## Discussion and learning points

Transitional cell carcinoma of the bladder remains the most prevalent histological subtype of bladder cancer, constituting an estimated 90% of bladder cancer diagnoses, with bladder adenocarcinoma constituting only 0.5%–2% of diagnoses (1, 7). As a consequence of the rarity of bladder adenocarcinoma, discussions of different treatment options are scarce in the literature; however, different management options for bladder adenocarcinoma have been studied, focusing on modalities such as radical cystectomy, radiotherapy, chemotherapy, combination therapies, and no treatment (8). Recent discussions have come to recognise that patients with urachal adenocarcinomas and bladder diverticulum tumours can be optimal candidates for partial cystectomy (9). Ashley et al. demonstrated in a review of 46 patients who underwent partial cystectomies and 14 patients who underwent radical cystectomies for urachal adenocarcinomas that there was no difference in survival between patients who underwent partial cystectomy vs. radical cystectomy (10). Furthermore, in a review by Dursun et al. of 841 patients with urachal carcinoma of the bladder, of whom ∼50% had >T2 disease, in local and locally advanced disease, there was no difference in survival between patients who underwent partial vs. radical cystectomy (HR: 1.75; 95% CI: 0.72–4.3) (11).

The benefits of offering partial cystectomy to patients have been well-documented and include bladder and sexual preservation and negating the need for urinary diversion (12). Additionally, partial cystectomy still allows patients to be accurately staged and even preserves the option of salvage cystectomy if disease recurrence occurs (9).

The use of near-infrared guidance in robotic surgery has been commonly discussed in the literature with respect to the simultaneous use of fluorescence with indocyanine green (ICG) dye to visualise tissue perfusion; however, the sole use of near-infrared imaging to transilluminate deep tissues in urological surgery has minimal coverage in the literature (13). One case report by Kurobe et al. discusses a similar technique to the one we have described, using near-infrared imaging with the da Vinci Firefly technology and transvesical illumination to excise a urachal cyst (5). The technique used by Kurobe et al. was very similar to that described in this report, as they too did not use ICG intraoperatively and solely relied upon the transillumination of the urachal lesion; however, this was for benign disease, unlike the case reported here. Therefore, the technique described in this case report is novel, as it is the first to specifically describe oncological resection of a urachal tumour using near-infrared imaging, without the use of ICG.

This report demonstrates how using near-infrared imaging for partial cystectomy can enhance intraoperative identification of the tumour and its margins. Similar to other case reports in this field, we demonstrated how the additional use of the Firefly system allows for real-time visualisation of tumour boundaries.

The advantages of near-infrared imaging without ICG include logistical benefits such as not having to dose the patient multiple times with ICG, reduced dependence on variable tissue perfusion or tissue uptake of ICG when operating, reduced costs, and reduced risk of anaphylaxis (in cases of iodine/shellfish allergies). The disadvantages of using near-infrared imaging without ICG include varied light wavelength penetration depending on tissue depth/thickness and subjective interpretation due to the use of anatomical boundaries rather than molecular dye boundaries. Additionally, near-infrared imaging without ICG is a more novel technique and is less established in the literature, whereas the intraoperative use of ICG has a more extensive evidence base.

We conclude that intraoperative near-infrared guidance may support the confident surgical resection of tumour margins and aid in the preservation of healthy bladder tissue. While this technique offers a promising option for surgeons to confidently perform oncological resections in partial cystectomies, careful patient selection when performing bladder-sparing surgeries is of paramount importance. Additionally, further studies, including randomised controlled trials, are required to determine the long-term oncological outcomes for patients undergoing near-infrared-guided robotic partial cystectomy for bladder adenocarcinoma using the Firefly platform compared to other treatment modalities, such as radical cystectomy, radiotherapy, chemotherapy, combination therapies, and no treatment.

## Data Availability

The original contributions presented in the study are included in the article/Supplementary Material, further inquiries can be directed to the corresponding author.
